# The Role of Neutrophil-Lymphocyte Ratio in Identifying the Severity of Obstructive Sleep Apnea: A Retrospective Study

**DOI:** 10.7759/cureus.99814

**Published:** 2025-12-22

**Authors:** Muntadher H Jasim, Abdullah S Kadhim, Ameer A Al-Musawi, Ali S Msaede, Alyaa A Al-Masoodi, Mohammed N Taha

**Affiliations:** 1 Internal Medicine, Dhari Fayadh Teaching Hospital, Baghdad, IRQ; 2 Internal Medicine, Al-Shaheed Sadr Hospital, Baghdad, IRQ; 3 Internal Medicine, Al-Shaab Hospital, Baghdad, IRQ; 4 Internal Medicine, Al-Numan Teaching Hospital, Baghdad, IRQ; 5 Rheumatology, Al-Kindy College of Medicine, Baghdad, IRQ; 6 Internal Medicine, Baptist Health Medical Center-Little Rock, Arkansas, USA

**Keywords:** apnea–hypopnea index, cutoff scores, neutrophil to lymphocyte ratio, obstructive sleep apnea - osa, polysomnography

## Abstract

Background: Obstructive sleep apnea (OSA) is a prevalent disorder characterized by recurrent episodes of upper airway blockage during sleep, leading to intermittent hypoxia and disturbed sleep patterns. It is often associated with systemic inflammation as well as metabolic and cardiovascular disorders. The neutrophil-to-lymphocyte ratio (NLR) has emerged as a promising predictor of systemic inflammation. This study aimed to evaluate the role of NLR in assessing the severity of OSA.

Patients and methods: This retrospective cross-sectional study included 100 adult patients diagnosed with OSA through overnight polysomnography at a sleep center in Baghdad, Iraq, from April 6 to October 3, 2025. Patients with active infections, chronic inflammatory or autoimmune diseases, malignancy, or hematologic disorders were excluded. The severity of OSA was classified based on the apnea-hypopnea index (AHI). Statistical analysis was performed using SPSS software (IBM Corp., Armonk, NY), with t-tests and Fisher’s exact tests applied and receiver operating characteristic (ROC) analysis used to determine the diagnostic efficiency of NLR.

Results: Among the 100 patients, 42% had mild OSA, 25% moderate OSA, and 33% severe OSA. Body mass index (BMI) was significantly higher among patients with severe OSA (p = 0.025). ROC analysis revealed that NLR significantly predicted severe OSA (area under the curve (AUC)= 0.760, p < 0.001). An optimal cut-off point of NLR = 3.6 achieved 78.7% sensitivity, 74.6% specificity, 94.1% negative predictive value (NPV), and 87.7% positive predictive value (PPV).

Conclusion: The NLR shows promise as an accessible and cost-effective biomarker for identifying severe OSA. Elevated NLR values reflect the systemic inflammatory processes associated with intermittent hypoxia and may aid clinicians in assessing OSA severity, particularly when polysomnography is not readily available.

## Introduction

According to the American Academy of Sleep Medicine, obstructive sleep apnea (OSA) is described in the International Classification of Sleep Disorders, Third Edition, as a condition confirmed by polysomnography showing an obstructive respiratory disturbance index of five or more events per hour when accompanied by symptoms such as excessive daytime sleepiness, fatigue, insomnia, or loud snoring. It may also be diagnosed when the index reaches 15 or more events per hour, even in the absence of these symptoms [1]. It has been found to be associated with a range of comorbid conditions, including hypertension, obesity, diabetes mellitus, gastroesophageal reflux disease, hypercholesterolemia, asthma, and serious cardiovascular diseases such as ischemic heart disease and heart failure. Additionally, OSA increases the risk of stroke, cognitive dysfunction, and metabolic disorders [2].

Individuals with suspected OSA typically exhibit severe daytime somnolence, pronounced snoring, episodes of gasping or choking, or observed instances of breathing pauses during sleep. Excessive daytime somnolence is among the most prevalent symptoms. A significant number of patients may just express daytime fatigue, without accompanying symptoms [3].

The gold standard for evaluating the severity of OSA is nighttime in-laboratory level 1 polysomnography (PSG). PSG provides comprehensive data on apnea and hypopnea events during sleep, from which the apnea-hypopnea index (AHI) is calculated. The AHI is defined as the average number of apnea and hypopnea events per hour of sleep, which is commonly used to classify OSA severity as mild (5-15 events/hour), moderate (15-30 events/hour), or severe (>30 events/hour) [4,5].

OSA is more common in males. A study conducted by Benjafield et al. assessed the global prevalence of this condition, utilizing credible prevalence data from 16 countries, including the United States, China, Australia, and India. The global prevalence of OSA was derived from these data, indicating that over one billion individuals aged 30 to 65 are afflicted by OSA, with 425 million classified as having moderate to severe OSA [6].

OSA pathophysiology involves recurrent upper airway collapse during sleep due to a combination of anatomical and functional factors. Anatomically, the upper airway becomes narrowed from excess soft tissue, craniofacial abnormalities, or fluid shifts, leading to increased airway collapsibility. Functionally, during sleep, there is a reduction in the neuromuscular tone of the upper airway dilator muscles, particularly the genioglossus, which impairs the airway's ability to stay open against negative inspiratory pressures. This collapse causes apnea or hypopnea episodes, resulting in intermittent hypoxia, hypercapnia, and sleep fragmentation due to recurrent arousals [7,8].

Emerging evidence suggests that systemic inflammation, alongside mechanical variables such as upper airway collapsibility, contributes to the etiology of OSA. Histologic abnormalities, including subepithelial edema and increased infiltration of inflammatory cells in human bronchial epithelial cells, have been documented [9].

Recently, many indicators of systemic inflammation derived from standard blood tests have garnered interest due to their accessibility and affordability. The neutrophil-to-lymphocyte ratio (NLR) is acknowledged as a dependable indicator of systemic inflammation with prognostic significance in several chronic disorders, presumably due to continuous systemic inflammation stimulating white blood cells throughout disease progression [10].

This study aimed to evaluate the role of the NLR, obtained from routine complete blood count, in identifying the severity of OSA among adult patients diagnosed by polysomnography in Baghdad, Iraq.

## Materials and methods

This retrospective cross-sectional study was conducted in Baghdad/ Iraq. The data was collected during the period from April 6 to October 3, 2025. A total of 127 consecutive patients (inpatients and outpatients) diagnosed with OSA via overnight polysomnography in Baghdad during the given time period were assessed for eligibility. A total of 100 patients were included. The inclusion criteria were adult patients (aged 18 years and above) diagnosed with OSA through overnight polysomnography. Included participants had complete clinical and laboratory data, including neutrophil and lymphocyte counts, and no prior treatment for OSA.

**Figure 1:**
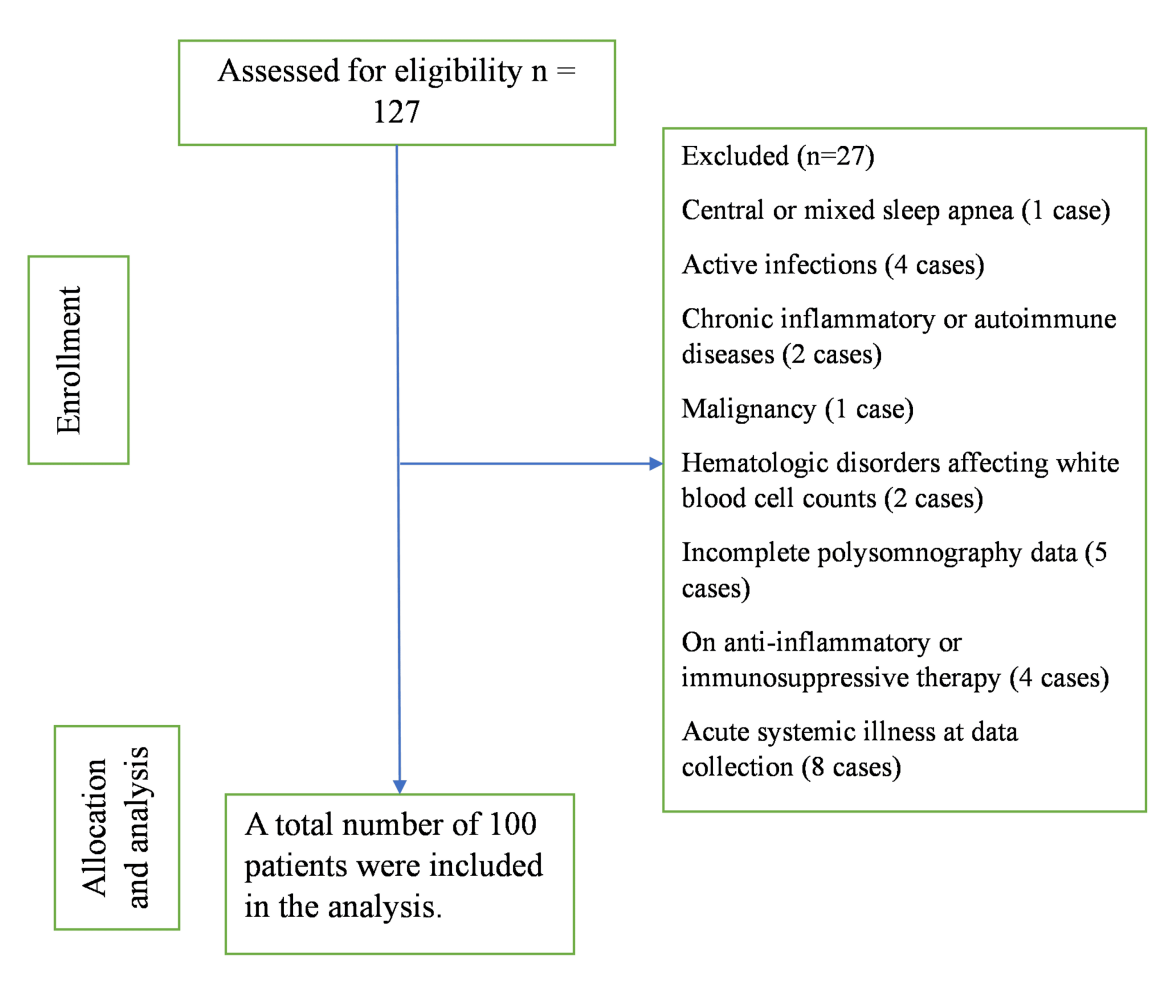
Cohort flowchart of patient inclusion and exclusion

The exclusion criteria were patients with central or mixed sleep apnea (one case), active infections (four cases), chronic inflammatory or autoimmune diseases (two cases), malignancy (one case), or hematologic disorders affecting white blood cell counts (two cases). Patients with incomplete polysomnography data (five cases), those on anti-inflammatory or immunosuppressive therapy (four cases), or with acute systemic illness (eight cases) at the time of data collection were also excluded, as shown in Figure 1.

Ethical consideration

All participants provided written permission prior to the collection of data. A formal permission letter was secured from an Institutional Review Board in compliance with the Declaration of Helsinki.

Data collection

After obtaining consent, patient data were collected, including age, sex, weight, height, and CBC counts. Blood samples for CBC were obtained routinely at the time of OSA diagnostic evaluation (on the same day or within one to two days of polysomnography). Samples were collected in ethylenediaminetetraacetic acid (EDTA) tubes and processed within two hours using an automated hematology analyzer (Sysmex XN-1000), with neutrophil and lymphocyte counts reported as ×10⁹/L. Overnight polysomnography was performed using Nox-T3 portable PSG systems (Nox Medical, Iceland), calibrated according to manufacturer guidelines, and recorded EEG, EOG, EMG, airflow, respiratory effort, oximetry, and heart rate. The diagnosis of OSA was based on an AHI ≥ 5. Severity was classified according to the American Academy of Sleep Medicine guidelines as mild (5-15 events/hour), moderate (15-30 events/hour), or severe (>30 events/hour) [5].

Statistical analysis

Data analysis was conducted using SPSS software (version 26; IBM Corp., Armonk, NY). Mild and moderate OSA were grouped for comparison with severe OSA to maximize statistical power (n = 67 vs n = 33). To compare continuous variables, the independent samples t-test was applied, while Fisher's exact test was utilized for categorical data. Results with a two-tailed p-value of 0.05 or less were considered statistically significant. ROC analysis was conducted to evaluate the role of the neutrophil-to-lymphocyte ratio (NLR) for the prediction of severe OSA.

## Results

**Figure 2:**
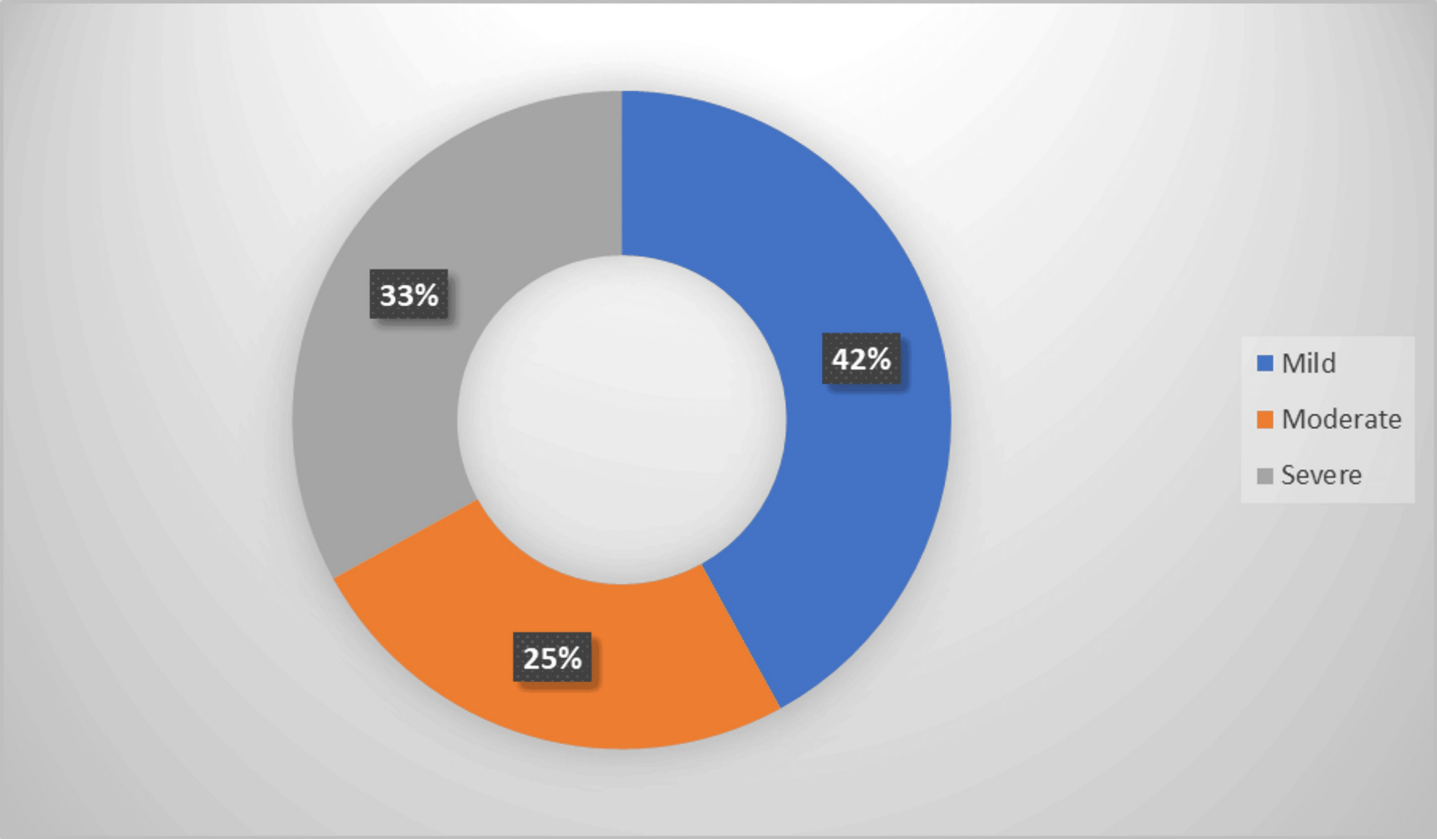
Distribution of the studied sample according to OSA severity OSA: Obstructive sleep apnea.

Among the 100 OSA patients included in this study, 42 (42.0%) had mild OSA, 25 (25.0%) had moderate OSA, and 33 (33.0%) had severe OSA, as shown in Figure 2.

Table 1 shows no statistically significant difference in age between the groups, with mean ages of 50.39 ± 11.41 and 51.75 ± 12.20 years (p = 0.596). Body mass index (BMI) was significantly higher in the severe OSA group (32.21 ± 4.51) compared to the mild/moderate group (30.12 ± 4.18), with a p-value of 0.025. In addition, patients with severe OSA exhibited markedly higher neutrophil counts (6.91 ± 2.27 × 10⁹/L) and NLRs (5.42 ± 3.09) compared with those with mild/moderate OSA (5.04 ± 2.16 × 10⁹/L and 2.74 ± 2.20, respectively). Conversely, lymphocyte counts were significantly lower in the severe OSA group (1.62 ± 0.83 × 10⁹/L) than in the mild/moderate group (2.39 ± 0.95 × 10⁹/L).

**Table 1:** Comparison of basic and characteristics between patients with severe obstructive sleep apnea and those with mild/moderate obstructive sleep apnea Data are presented as mean ± SD for continuous variables, such as age, BMI, neutrophil, lymphocyte, and NLR. * An independent sample t-test was used for statistical analysis.

Parameters	Severe OSA (n = 33)	Mild/Moderate OSA (n = 67)	P-value
Age (years)	50.39 ± 11.41	51.75 ± 12.20	0.596^*^
BMI (kg/m²)	32.21 ± 4.51	30.12 ± 4.18	0.025^*^
Neutrophil (×10^9^/L)	6.91 ± 2.27	5.04 ± 2.16	<0.001^*^
Lymphocyte (×10^9^/L)	1.62 ± 0.83	2.39 ± 0.95	<0.001^*^
NLR	5.42 ± 3.09	2.74 ± 2.20	<0.001^*^

Regarding sex distribution, the severe OSA group (n = 33) included 18 males (54.5%) and 15 females (45.5%), while the mild/moderate OSA group (n = 67) included 39 males (58.2%) and 28 females (41.8%). There was no statistically significant difference in sex distribution between the groups (p = 0.831, Fisher’s exact test).

Efficiency of NLR for the identification of severe OSA

**Figure 3:**
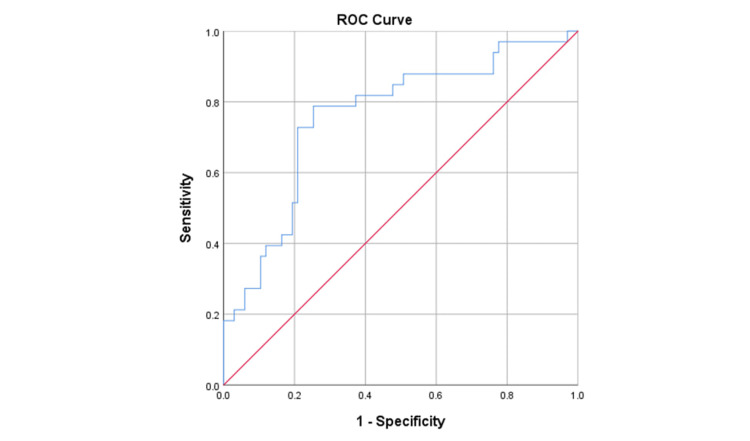
ROC analysis of the diagnostic indices of NLR for identifying severe OSA ROC: Receiver operating characteristic; NLR: Neutrophil-to-lymphocyte ratio; OSA: Obstructive sleep apnea.

The ROC analysis revealed that the NLR was a significant predictor of severe OSA (area under the curve (AUC) = 0.760, P-value < 0.001), as shown in Figure 3.

The optimum cut-off point with the highest (sensitivity + specificity) was determined to be 3.6, as it had a sensitivity of 78.7%, specificity of 74.6%, positive predictive value (PPV) of 60.4%, and negative predictive value (NPV) of 87.7%, as shown in Table 2.

**Table 2:** Efficiency of 3.6 NLR as a cut-off point for the identification of severe OSA Data are presented as n (number of patients). NLR: Neutrophil-to-lymphocyte ratio; OSA: Obstructive sleep apnea.

NLR	Group		Total
	Severe OSA	Mild/moderate OSA	
≥3.6	26	17	43
<3.6	7	50	57
Total	33	67	100

## Discussion

OSA is a prevalent disorder with considerable detrimental effects. This illness may remain unrecognized and untreated in over 80% of cases [11]. Polysomnography is the most definitive method for diagnosing OSA; however, it necessitates specialized technical expertise, is considered expensive, and is not easily accessible [12]. Hence, there is a need for a more straightforward and cost-effective strategy. Evaluating the severity of OSA is crucial for determining the right treatment approach, including conservative therapy for mild cases and more intensive therapies, such as continuous positive airway pressure (CPAP), for severe cases [13].

The current study found that 42% of patients had mild OSA, 25% had moderate OSA, and 33% had severe OSA. A study conducted by Nokes et al. reported that mild OSA constituted the largest proportion (43%), followed by moderate (29%) and severe (28%) cases [14]. Sönmez et al. found that the distribution of OSA severity was severe (18%), moderate (30%), and mild (52%) [15].

The present study also found that higher BMI was a significant predictor of severe OSA. This finding is in concordance with the study by Fattal et al., who demonstrated that higher BMI was significantly associated with a higher AHI, indicating more severe OSA [16]. A study by Halima et al. found that the severity of OSA had a significant positive correlation with BMI [17], and Kuna et al. found that higher BMI was significantly associated with severe OSA [18]. It is suggested that excess fat deposition in the neck and surrounding upper airway leads to increased mechanical load and airway collapsibility during sleep. This results in a greater obstruction of airflow and more frequent apnea and hypopnea events, explaining why higher BMI is a significant risk factor for severe OSA [19].

In the present study, the optimal NLR cut-off point was 3.6, with a sensitivity of 78.7%, specificity of 74.6%, PPV of 87.7%, and NPV of 94.1%. Jiang et al. found that the best cut-off value of NLR was 4.27, with a specificity of 86.1% and sensitivity of 65.1%; PPV was 71%, and NPV was 82.2% [20]. The best cut-off level for N/L ratio was determined to be 4.1 with a sensitivity of 81.5 and a specificity of 72%, according to research conducted by Shetty et al. [21]. Altintas et al. found that the cut-off level for neutrophil-lymphocyte ratio was 1.85 with sensitivity and specificity of 85% and 71%, respectively [22]. Hatice et al. found that with a sensitivity of 58.8% and a specificity of 51.0%, an NLR of 2.40 or above indicated the likelihood of OSA [23]. Sunbul et al. reported that the sensitivity and specificity of NLR in detecting OSA were 56.2% and 63.1%, respectively [24].

Systemic inflammation and immune dysregulation may be induced by intermittent hypoxia, as recurrent airway obstruction in OSA leads to chronic oxygen desaturation and reoxygenation cycles, which activate oxidative stress pathways and stimulate nuclear factor-κB (NF-κB) signaling, leading to the secretion of cytokines that promote inflammation and thereby neutrophil activation and proliferation while suppressing lymphocyte counts. Conversely, pre-existing inflammation manifested as subepithelial edema and inflammatory cell infiltration may exacerbate airway collapsibility. The resultant elevation in NLR indicates an inflammatory shift toward neutrophil-mediated tissue injury and reduced adaptive immune regulation, both of which participate in vascular endothelial dysfunction, free radicals, and cardiovascular risk associated with severe OSA.

This study has several limitations. First, it employed a retrospective design. Second, although the sample size (n = 100) provided adequate power for primary analyses, it may have limited the detection of subgroup effects. Third, unmeasured confounders, such as smoking status and detailed comorbidities, could influence the associations, although the exclusion criteria minimized acute inflammatory biases. Finally, while rigorous exclusion criteria minimized acute confounders, undiagnosed chronic conditions could subtly elevate NLR, evading detection.

## Conclusions

The NLR shows promise as an accessible and cost-effective biomarker for identifying severe OSA. Elevated NLR values reflect the systemic inflammatory processes associated with intermittent hypoxia and can aid clinicians in assessing OSA severity, particularly when polysomnography is not readily available. Further studies on larger populations are recommended to validate these findings. It would also be interesting to explore the utility of NLR as a follow-up marker to evaluate treatment response in patients with OSA.
